# Therapeutic effect of intranasal nicotinamide adenine dinucleotide in the restoration of olfactory dysfunction

**DOI:** 10.1038/s12276-026-01761-9

**Published:** 2026-07-01

**Authors:** Shin Hyuk Yoo, Jung Yeon Jang, Jun-Sang Bae, Reiza Ventura, Eun Hee Kim, A Young Kim, Ji-hun Mo, Jaewoo Park, Kyuho Kang, Yeogyun Yun, Jun Hee Lee, Yong-Jae Kim, Dong-Joon Lee, Ji Heui Kim

**Affiliations:** 1 Department of Otorhinolaryngology, Dankook University College of Medicine, Cheonan, Republic of Korea; 2 Department of Otorhinolaryngology — Head and Neck Surgery, Asan Medical Center, University of Ulsan College of Medicine, Seoul, Republic of Korea; 3 Department of Biological Sciences and Biotechnology, Chungbuk National University, Cheongju, Republic of Korea; 4 Institute of Tissue Regeneration Engineering (ITREN), Dankook University, Cheonan, Republic of Korea; 5 Mechanobiology Dental Medicine Research Center, Dankook University, Cheonan, Republic of Korea; 6 Department of Biomaterials Science, College of Dentistry, Dankook University, Cheonan, Republic of Korea; 7 Department of Oral Histology, College of Dentistry, Dankook University, Cheonan, Republic of Korea

**Keywords:** Drug development, Neurodegeneration

## Abstract

Olfactory dysfunction is a debilitating condition with no established treatment. This study evaluated the efficacy of intranasal (i.n.) NAD administration in restoring olfactory function. Cultured human olfactory stem cells (hOSCs) were treated with NAD and assessed by immunofluorescence staining, PCR, and western blot analyses. In vivo, mice with ZnSO_4_-induced anosmia were treated with i.n. NAD, intraperitoneal dexamethasone, or PBS and evaluated by histological analysis, behavioral tests, bulk RNA-sequencing (RNA-seq), and in situ hybridization. NAD promoted hOSC differentiation into olfactory sensory neurons (OSNs), evidenced by increased stem cell (SOX2 and nestin) and OSN markers (Tuj1 and OMP) expression, and upregulated neuronal differentiation-related genes (*SOX2, NESTIN, NEUROD1, NEUROG1*, and *OMP*). In vivo, the NAD group showed significant olfactory function improvement and marked olfactory epithelium repair. Bulk RNA-seq of the olfactory turbinate tissue identified 113 differentially expressed genes (cluster T1) upregulated in control and NAD groups. The Gene Ontology (GO) term “modulation of chemical synaptic transmission” was associated with cluster T1, and 25 genes implicated in this GO were upregulated in the NAD group. Integration with publicly available single-cell RNA-seq data identified six neuronal marker genes — *ABHD2, DLGAP2, FOXO3, HIPK2, KCNMA1*, and *PCDH17* — upregulated by NAD. Protein expression of DLGAP2 and PCDH17 was higher in differentiated hOSCs treated with NAD. In situ hybridization confirmed that *Dlgap2*, *Foxo3*, and *Pcdh17* expression was restored in anosmic mice treated with i.n. NAD. The potential therapeutic efficacy of i.n. NAD administration was demonstrated by showing regeneration of OSNs in hOSCs and restoring olfactory function in an anosmia mouse model.

## NAD treatment promotes olfactory neuron regeneration

Olfactory dysfunction affects a significant portion of the global population, impacting quality of life and increasing mortality risk. Current treatments, including surgery and steroids, offer limited effectiveness. This study explores the potential of NAD to enhance olfactory function by promoting the differentiation of human olfactory stem cells into olfactory sensory neurons. Researchers treated human olfactory stem cells with NAD and assessed differentiation through immunofluorescence and gene expression analyses. Results showed that NAD increased the expression of neuronal markers, suggesting enhanced neurogenesis and maturation of olfactory sensory neurons. In an anosmia mouse model, intranasal NAD administration significantly improved olfactory function and restored the olfactory epithelium. These findings highlight NAD’s potential as a therapeutic agent for olfactory dysfunction, suggesting that future research could explore its clinical applications in treating this condition. This summary was initially drafted using artificial intelligence, then revised and fact-checked by the author.

## Introduction

Olfactory dysfunction, estimated to affect 3–20% of the worldwide population, substantially impacts individual well-being and quality of life and can increase mortality risk by up to four times^1–4^. The treatment approach for olfactory dysfunction depends on its underlying cause and may include surgery, olfactory training, and oral or intranasal (i.n.) steroid sprays^5–7^. However, the management of this condition is challenging, given that the effectiveness of these treatments is not guaranteed^8^.

NAD is a coenzyme continuously produced, consumed, and recycled within cells, allowing for dynamic regulation. Beyond its role in redox reactions^9^, NAD is crucial for various physiological processes such as energy metabolism, transcriptional regulation, and DNA repair^10–12^. It also influences pathological processes linked to inflammation, cancer, and neurodegeneration^13^. Recent research indicates that NAD deficiency is associated with inflammation, poor DNA repair, and mitochondrial dysfunction, whereas increased NAD levels enhance metabolic fitness^14^. Numerous studies have demonstrated the therapeutic potential of NAD in neurological disorders^14,15^. Given these insights, NAD may have the potential to manage olfactory dysfunction caused by injury to the olfactory sensory neurons (OSNs) of the olfactory epithelium (OE), although no studies have specifically explored this application.

In this study, we treated human olfactory stem cells (hOSCs) with NAD to determine whether NAD facilitates their differentiation into OSNs. We also aimed to investigate whether i.n. delivery of NAD could enhance the restoration of olfactory dysfunction in an anosmia mouse model. Behavioral tests and histological analyses were performed to assess the restoration of olfactory function and regeneration of the OE and further mechanistic insights were obtained.

## Materials and methods

### Collection of olfactory mucosa and isolation of OSCs in humans

The collection of human olfactory mucosa and isolation of hOSCs were performed following the protocol described by ref. ^16^. Nasal cavities were examined using a 0° or 30° rigid endoscope. The olfactory mucosa was harvested using ethmoid forceps to obtain a 2 mm^2^ tissue sample from either the root of the medial aspect of the middle turbinate or the dorsomedial area of the septum. The olfactory mucosa was rinsed with DMEM/HAM F12 (Gibco, Thermo Fisher Scientific, Waltham, MA, USA) and incubated in 2.4 IU/ml dispase II solution (STEMCELL Technologies, Vancouver, Canada) at 37 °C for 1 h to facilitate tissue separation. The olfactory mucosa was dissected from the underlying lamina propria using a microspatula under a dissecting microscope (Olympus, Tokyo, Japan). Differential characteristics such as the translucent appearance of the epithelium against a black background and striped, orange/brown lamina propria were noted. The lamina propria were then transferred to a Petri dish containing DMEM/HAM F12 and sliced into pieces (200–500 μm thickness). These pieces were placed in 2 cm diameter culture dishes, covered with sterile glass coverslips, and supplemented with 500 μl of culture medium (DMEM/HAM F12 supplemented with 10% fetal bovine serum (FBS) and antibiotics). The medium was renewed every 2–3 days. Stem cell invasion began within 5–7 days, and confluency was achieved after 2 weeks. Cells were passaged and transferred to culture flasks. This study was conducted in accordance with the Declaration of Helsinki and approved by the Institutional Review Board of Dankook University Hospital (IRB No. 2024-05-014).

### Cell viability assay of NAD for hOSCs

Cell viability assay was performed to determine optimal in vitro NAD (Sigma-Aldrich, St Louis, MO, USA) concentration. hOSCs within five passages were seeded at 1 × 10^6^ cells/ml in a 96-well plate containing 50 μl/well of DMEM/F12 with 10% FBS. Cells were treated with NAD stock solution (50 mM in DMEM/F12 within 10% FBS) at final concentrations of 0–20 mM. After 24 h of culture in a CO_2_ incubator, 10 μl/well of CCK-8 reagent (DOJINDO Laboratories, Kumamoto, Japan) was added. After a 2-h incubation, optical density (OD) was measured at 450 nm using a microplate reader (Thermo Fisher Scientific). The OD of cells at 0 mM NAD was considered at 100% cell viability, and the relative viability of cells treated with other NAD concentrations was calculated.

Two sets of NAD concentrations were tested: 0–20 mM and 0–10 mM (Supplementary Fig. 1). No significant differences in cell viability were observed, but NAD concentrations between 2.5 mM and 5.0 mM increased hOSCs viability the most. Therefore, 2.5 mM NAD and 5.0 mM NAD were used for subsequent experiments.

### Neuronal differentiation of hOSCs

Neuronal differentiation of hOSCs was induced using Neurobasal Medium^TM^ (Gibco, Thermo Fisher Scientific) and DMEM/F12 medium supplemented with B-27, L-glutamine (GlutaMAX, Gibco, Thermo Fisher Scientific), glutamate (2 mM), penicillin (50 U/ml), and streptomycin (50 μg/ml). To evaluate the effect of NAD on neuronal differentiation, hOSCs were incubated with differentiation media containing or not containing 2.5 mM or 5.0 mM NAD, with the medium changed every 3 days.

### Immunofluorescent staining and tissue analysis using confocal microscopy

hOSCs were fixed with 4% paraformaldehyde, washed with PBS, permeabilized with 0.5% Triton X-100, and then blocked with 5% bovine serum albumin (Sigma-Aldrich). Subsequently, cells were incubated with primary antibodies, anti-SOX2 for pluripotent neural stem cells (1:100; Abcam, Cambridge, UK, cat. no. ab97959), anti-nestin for neuronal stem cell marker (1:100; Abcam, cat. no. ab22035), anti-Tuj1 for immature OSNs (1:500; BioLegend, San Diego, CA, USA, cat. no. 801202), anti-OMP for mature OSNs (1:100; Thermo Fisher Scientific, cat. no. PA5-115686), anti-DLGAP2 (1:100, Novus Biologicals, Centennial, CO, USA, cat. no. NBP2-82086), and anti-PCDH17 (1:50, Novus Biologicals, cat. no. NBP2-97901) overnight at 4 °C. Cells were then washed with PBS and incubated with Alexa Fluor 488 goat anti-mouse (1:500; Invitrogen, Thermo Fisher Scientific) or Alexa Fluor 594 goat anti-rabbit (1:500; Invitrogen, Thermo Fisher Scientific) secondary antibodies for 1 h at 25 °C. Cell nuclei were counterstained with 4′,6-diamidino-2- phenylindole (DAPI; Sigma-Aldrich, cat. no. D9542). Cells were observed and imaged under a confocal microscope (FV-3000; Olympus) at 400× magnification.

Signal intensity of individual cells was quantified using ImageJ software (National Institutes of Health, Bethesda, MD, USA). Fluorescence images were separated into individual channels and converted to 16-bit grayscale format. Threshold parameters were determined based on control group images and applied uniformly across all experimental groups to minimize background signal. Regions of interest (ROIs) were manually delineated around individual cells, and mean fluorescence intensity was measured using the Analyze function in ImageJ. All mean fluorescence intensity values are expressed in arbitrary units.

### PCR analyses

Quantitative real-time PCR was performed using the RIboEx^TM^ reagent (GeneAll Hybrid R Kit, GeneAll Biotechnology Co., Ltd, Seoul, Republic of Korea) to extract total RNA from hOSCs treated with NAD for 1 week, following the manufacturer’s instructions. RNA was reverse-transcribed using an iScript cDNA Synthesis Kit (Gene All Biotechnology Co., Ltd). mRNA expression was analyzed using an Applied Biosystems 7500 Real-Time PCR System (Applied Biosystems, Waltham, MA, USA).

The relative mRNA expression levels of *DLGAP2, FOXO3, HIPK2, PCDH17, SOX2, NESTIN, NEUROG1, NEUROD1, OMP*, and *GAPDH* were evaluated using SYBR Green primers (Bioneer, Daejeon, Republic of Korea) and Applied Biosystems (Supplementary Table 1). Relative mRNA expression was calculated using the 2^−ΔΔ^Ct method and normalized to *GAPDH*.

### Western blot analysis

Cells were lysed in radioimmunoprecipitation assay buffer (Thermo Fisher Scientific) supplemented with Halt phosphatase and protease inhibitor cocktail (Thermo Fisher Scientific). Total cell lysates (20–50 µg per well) were resolved by 10–12% SDS–PAGE and transferred to nitrocellulose membranes (Amersham™ Protran, Cytiva, Marlborough, MA, USA). Following blocking with 5% bovine serum albumin in Tris-buffered saline containing 0.1% Tween-20, membranes were incubated with primary antibodies against SOX2 (1:1000, Cell Signaling Technology, Danvers, MA, USA, cat. no. 23064), nestin (1:1000, Sigma-Aldrich, cat. no. MAB353), and OMP (1:1000, GeneTex, Irvine, CA, USA, cat. no. GTX04312). Membranes were then incubated with appropriate horseradish peroxidase-conjugated secondary antibodies (Cell Signaling Technology). Horseradish peroxidase-conjugated anti β-actin (1:2000, Cell Signaling Technology, cat. no. 5125) was used as a loading control. Immunoreactive bands were visualized using SuperSignal West Pico Plus Chemiluminescent Substrate (Thermo Fisher Scientific), and images were captured and analyzed using the iBright C1500 system and iBright Analysis Software (Thermo Fisher Scientific).

### Anosmia mouse model

Forty female BALB/c mice (6–7 weeks old, 18–20 g; Orient Bio Inc., Sungnam, Republic of Korea) were divided into four groups (*n* = 10 per group): control, ZnSO_4_-induced anosmia group, NAD i.n. treatment, and dexamethasone (DXM) intraperitoneal (i.p.) injection (Supplementary Fig. 2a).

Anosmia was induced by administering 20 μl ZnSO_4_ (170 μM) i.n. in each nostril (total 40 μl), three times a week for a total of 4 weeks. Control mice received i.n. PBS. Mice were housed in a specific pathogen-free biohazard containment facility. All experiments adhered to the National Institutes of Health Guide for the Care and Use of Laboratory Animals and were approved by the Institutional Animal Care and Use Committee of the Asan Institute for Life Sciences (2019-02-165).

### Intranasal NAD administration

The NAD group received 100 mg/kg/day NAD (Sigma-Aldrich) i.n. daily for 4 weeks from Monday to Friday, starting 1 week after anosmia induction, administered in the head-down position. Control and anosmia groups received i.n. PBS, whereas the DXM group received 1 mg/kg i.p. DXM once a week for 4 weeks. Systemic corticosteroid therapy — particularly DXM — is frequently used in the management of olfactory dysfunction owing to its anti-inflammatory properties and potential to facilitate in the regeneration of OSNs^17–19^. Accordingly, systemic DXM i.p. injection was selected as a treatment control group in the present study.

### Behavioral tests and tissue preparation

Food-finding behavioral tests were conducted weekly to assess olfactory function (Supplementary Fig. 2b). Induction and recovery from anosmia were assessed using a food-finding test. Mice were food-deprived for 1 day and then released into an open field (buried food test) and T-maze (T-maze test) for 3 min each to find buried food pellets. The food pellets were buried randomly under the sawdust on one side of the cage (buried food test) or one arm of the T-maze (T-maze test). Each test mouse started at the opposite corner of the pellet (buried food test) or at the base of the T (T-maze test) and then walked forward to find hidden food. The elapsed time to locate the food was recorded. Food-finding behavioral tests were performed three times for each mouse. Mice were euthanized via cervical dislocation 24 h after the olfactory tests on day 28 for tissue sample collection and histological analysis.

### Histological analysis of anosmia mouse model

Mouse heads were collected after euthanasia, fixed in 10% neutral buffered formalin, decalcified with 14% ethylenediaminetetraacetic acid for 3 weeks, and embedded in paraffin. To evaluate the overall structure of nasal mucosa, 4-µm-thick paraffin sections were deparaffinized in xylene, rehydrated in alcohol, and stained with hematoxylin and eosin (H&E) (BBC Biochemical, Dallas, TX, USA). Paraffin sections selected for histological evaluation were located at the level just anterior to the anterior border of the eyes. The nasal tissue sections were observed under a bright-field microscope (Olympus) at 400× magnification. The thickness of the OE on H&E-stained slides was measured to assess the degree of OE recovery in each mouse.

For confocal imaging of the mouse models, immunofluorescence staining was performed to evaluate the status of immature or mature OSNs. Nasal mucosa tissues were incubated with the respective primary antibodies: Tuj1 for immature OSNs (1:500) and OMP for mature OSNs (1:200) overnight at 4 °C. The tissues were then washed with PBS and incubated with Alexa Fluor 594 goat anti-rabbit (1:500) or Alexa Fluor 488 goat anti-mouse (1:500) secondary antibodies for 1 h at 25 °C. Cell nuclei were counterstained with DAPI. The sections were assessed under a confocal microscope (FV-3000; Olympus) at 400× magnification. The number of Tuj1^+^ and OMP^+^ cells per high power field (HPF) in OE was measured using the ImageJ software. In this study, all measurements of OE thickness, as well as the quantification of Tuj1^+^ and OMP^+^ cells per HPF, were consistently performed in the dorsomedial region of the OE on the nasal septum, where OSN axons project to the olfactory bulb. This anatomical region was selected based on established protocols for OE analysis^20–22^. This region was specifically chosen because it consistently exhibits well-defined OSNs with characteristic morphology and organization, making it an optimal site for evaluating changes in epithelial thickness and neuronal differentiation markers.

### Bulk RNA-sequencing and single-cell RNA-sequencing data analysis from murine olfactory turbinate tissues

To analyze the bulk RNA-sequencing (RNA-seq) data, the reads were mapped to the mm10 genome using STAR version 2.7.1a with default parameters^23^. Subsequently, the aligned reads were converted into tag directories using HOMER version 4.11.1 (ref. ^24^). Each file was quantified using the “analyzeRepeats” script in HOMER. Gene expression levels in each sample were normalized by calculating the transcripts per kilobase million. Differentially expressed genes (DEGs) were identified via DESeq2 analysis based on raw read counts^25^. The “getDifferentialExpression” command in HOMER was applied with criteria for significance defined as an adjusted *P*-value of <0.05, more than a twofold difference in expression levels, and an average Fragments Per Kilobase of transcript per Million mapped reads (FPKM) > 2.

Our bulk RNA-seq dataset was integrated with a publicly available single-cell RNA-sequencing (scRNA-seq) dataset (accession numbers GSE184117) from human olfactory mucosa biopsies of healthy donors^26^ to ensure compatibility and uniformity in tissue localization. Only cells in which mitochondrial RNA accounted for less than 10% of the total RNA content were included. The data were integrated and anchored using the top 10,000 highly variable features identified using the variance-stabilizing transformation method. Subsequently, uniform manifold approximation and projection analysis was conducted and a nearest neighbor graph was constructed using the “FindNeighbors“ function in R version 4.3.0 (The R Foundation for Statistical Computing, Vienna, Austria). The “FindClusters” function was used to identify cell clusters with the resolution parameter set to 0.5. Differential expression analysis used the “FindMarkers” and “FindAllMarkers” functions. Principal component analysis and clustering were conducted using Seurat in R.

### In situ hybridization (ISH) analysis

Before RNA isolation, we confirmed that the target genes are expressed in relevant neural tissues using the NCBI Nucleotide Database: NM_172910.3 (*Mus musculus* DLG-associated protein 2, Dlgap2 mRNA), NM_001376967.1 (*M. musculus* forkhead box O3, Foxo3 mRNA), and NM_001013753.2 (*M. musculus* protocadherin 17, Pcdh17 mRNA). All target genes are expressed in the cortex and frontal lobe of the adult mouse brain. Male C57BL/6 mice (12 weeks old) were sacrificed for whole brain excision and total RNA extraction. Specimens were fixed overnight in 4% paraformaldehyde in PBS and embedded in paraffin. For in situ hybridization, specimens were treated with 20 μg/ml proteinase K for 20 min at 37 °C.

Antisense RNA probes were labeled with Digoxigenin RNA Labeling Mix (Roche, cat. no. 11277073910). RNA primer synthesis was performed using a non-transfection method according to the protocol described in ref. ^27^. Primers were designed using the PrimerQuest Tool (Integrated DNA Technologies). Primer sequences for the Dlgap2, Foxo3, and Pcdh17 genes are as follows:

*Dlgap2*: forward (T7): 5ʹ-GAGA TAA TAC GAC TCA CTA TAG GG tccagcagaagaggacataga-3ʹ; reverse (T3): 5ʹ-GAGA CAA TTA ACC CTC ACT AAA GG tcacaggctgagcacttaac-3ʹ;

*Foxo3*: forward (T7): 5ʹ-GAGA TAA TAC GAC TCA CTA TAG GG cccgtggaacagaactctataac-3ʹ; reverse (T3): 5ʹ-GAGA CAA TTA ACC CTC ACT AAA GG ccgactcagaactggaaactac-3ʹ;

*Pcdh17*: forward (T7): 5ʹ-GAGA TAA TAC GAC TCA CTA TAG GG tacgaacagaggcggagata-3ʹ; reverse (T3): 5ʹ-GAGA CAA TTA ACC CTC ACT AAA GG gtgatgaggctggcgataaa-3ʹ.

Total cDNA synthesis was performed according to the manufacturer’s protocol (MaximeTM PCR PreMix (i-MAX II), cat. no. 25265). RNA probe synthesis was performed according to the manufacturer’s protocol (NEB, cat. nos M0378 and M0251S).

Following hybridization, sections were incubated with anti-digoxigenin AP conjugate (1:1,000 dilution, Roche, cat. no. 11093274910) at 4 °C overnight. For visualization of RNA probes, NBT/BCIP Color Development Substrate (Promega, cat. no. S3771) was used. The specimens were sectioned at 16 μm thickness, and at least 20 sections from each experimental group were examined.

Sections were observed under a bright-field microscope (Olympus) at 400× magnification. The expression levels of target genes were evaluated by measuring signal intensity within the OE using ImageJ software. Anatomically consistent regions were designated as fixed measurement spots on each tissue section slide and defined as ROI for quantitative analysis. These spot locations were standardized and maintained identically across all experimental groups. The positively stained area was segmented using color thresholding, and data were expressed as the percentage of the stained area per total ROI area (% area). Negative control probes were used to assess background staining, and positive control probes were used to verify RNA integrity in the decalcified tissue samples.

### Statistical analysis

All statistical analyses were performed using the GraphPad Prism 10 software (GraphPad Software, San Diego, CA, USA). Results are expressed as the mean ± standard error of the mean. One-way analysis of variance and Tukey test for multiple comparison were used to compare the results between the groups. Statistical significance was set at *P* < 0.05.

## Results

### NAD promoted the differentiation of hOSCs into OSNs in vitro

The potential of NAD administration to facilitate the differentiation of hOSCs into neuronal stem cells and both immature and mature OSNs was investigated under condition with and without NAD administration.

**Fig. 1:**
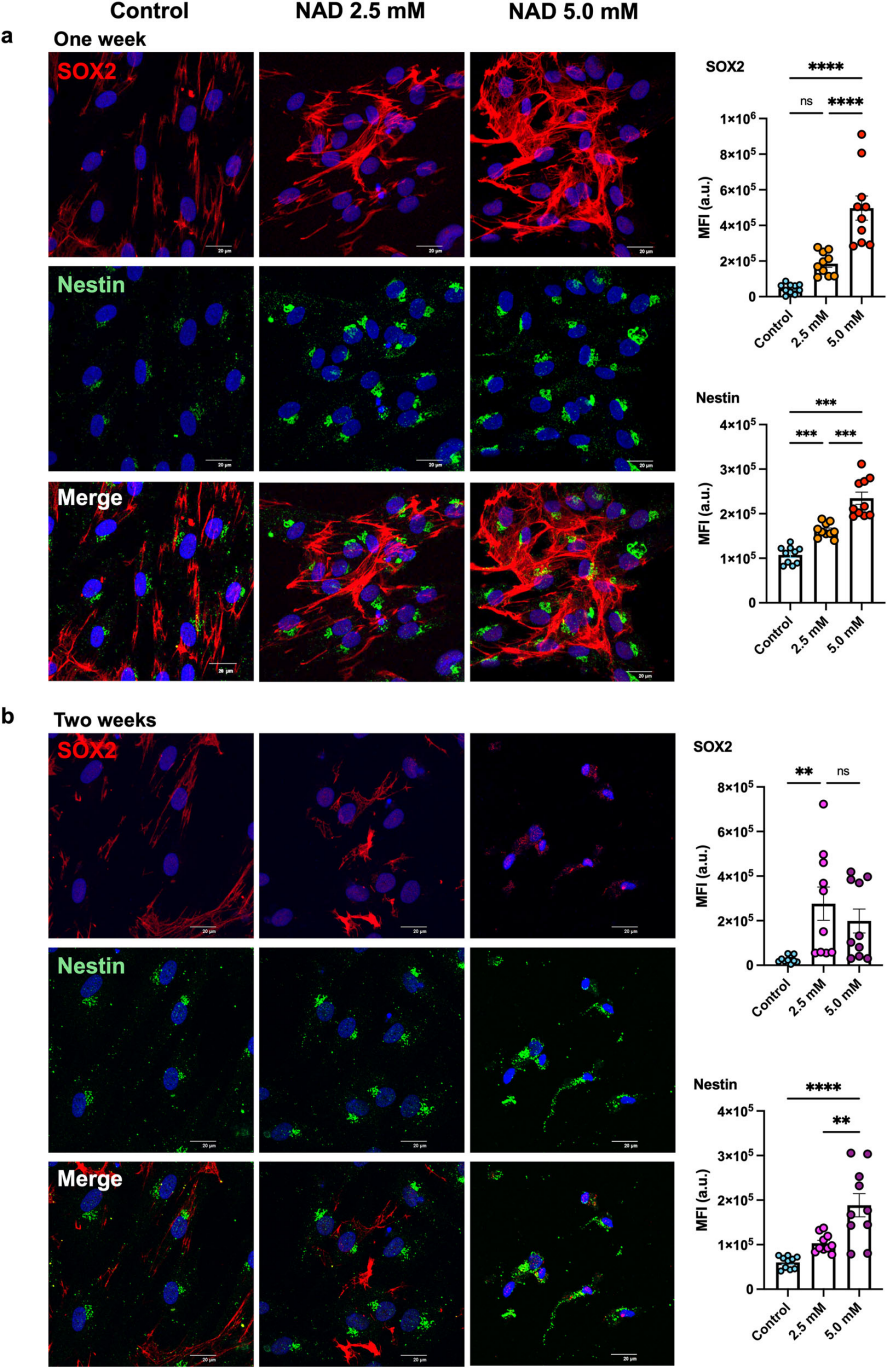
NAD enhances neuronal stem cell marker expression in human olfactory stem cells in vitro. **a**,**b**, Immunofluorescence staining showed that the intensities of SOX2^+^ and nestin^+^ markers were significantly increased in human olfactory stem cells treated with NAD (2.5 mM and 5.0 mM) compared with the control group, both at 1 week (part **a**) and 2 weeks (part **b**) post-neuronal differentiation. a.u., arbitrary unit; MFI, mean fluorescence intensity; ns, not significant. *P < 0.05, **P < 0.01, ***P < 0.001, ****P < 0.0001. *n* = 10 in each group. Scale bar, 20 µm.

**Fig. 2:**
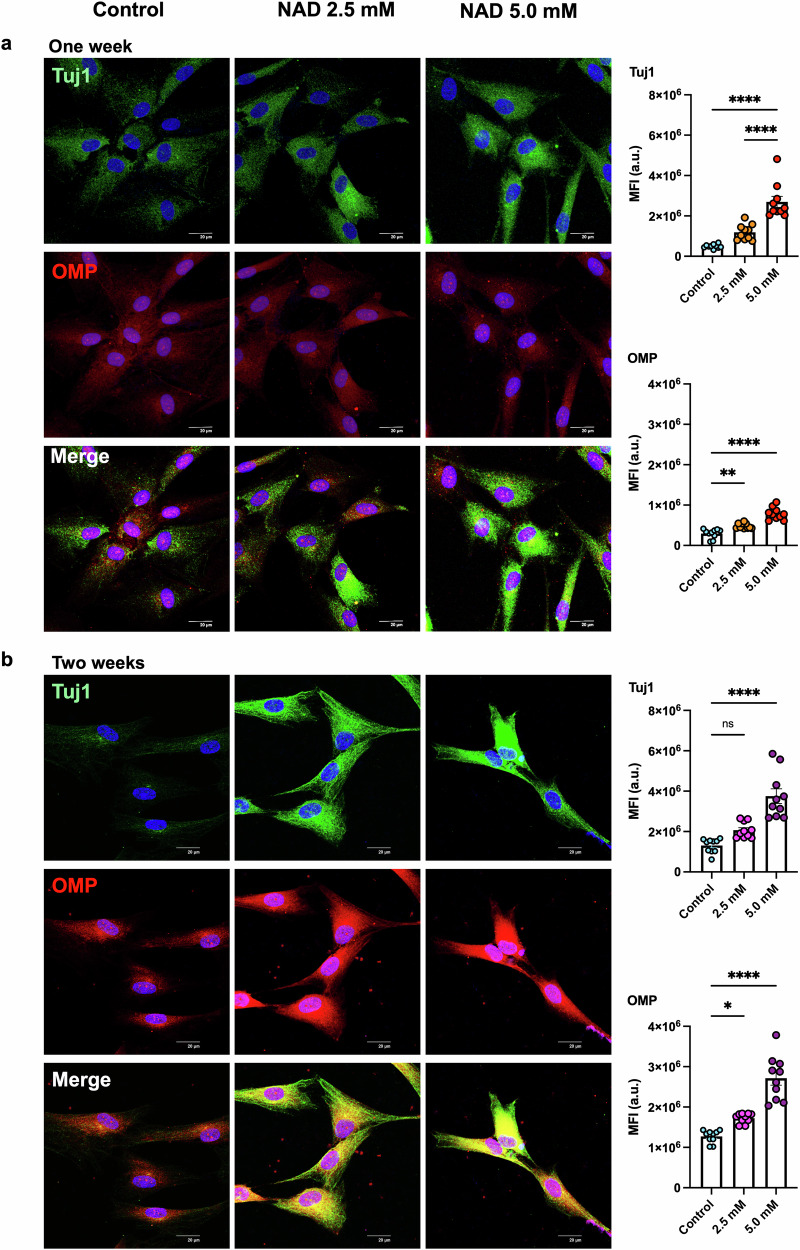
NAD promotes the differentiation of human olfactory stem cells into olfactory sensory neurons in vitro. **a**,**b**, Immunofluorescence staining revealed increased intensities of Tuj1^+^ (immature olfactory sensory neurons (OSNs)) and OMP^+^ (mature OSNs) markers in human olfactory stem cells treated with NAD (2.5 mM and 5.0 mM) compared with the control group at 1 week (part **a**) and 2 weeks (part **b**) post-neuronal differentiation. These results indicate that NAD promotes the differentiation of human olfactory stem cells into both immature and mature OSNs. a.u., arbitrary unit; MFI, mean fluorescence intensity. *P < 0.05, **P < 0.01, ***P < 0.001, ****P < 0.0001. *n* = 10 in each group. Scale bar, 20 µm.

First, the effect of NAD treatment on stem cell marker expression was analyzed via immunostaining of SOX2 and nestin, established neuronal stem cell markers, using confocal imaging analysis at 1 and 2 weeks after differentiation, respectively. SOX2 is known to be expressed not only in neural stem cells but also in various stem cell populations, including embryonic stem cells, where it performs a critical function in maintaining stemness. By contrast, nestin is recognized as a more neuronal stem cell-specific marker^27,28^. Compared with the control group, the mean intensities of SOX2 and nestin were significantly increased in the 2.5 mM and 5.0 mM NAD groups 1 week after differentiation (Fig. 1a). However, in the 5.0 mM NAD group 2 weeks after differentiation, the mean intensity of SOX2 was decreased, whereas that of nestin was increased (Fig. 1b). Second, in the analysis of OSN expression, compared with the control group, the mean intensity of Tuj1, representing immature OSNs, was significantly increased in the 2.5 mM and 5.0 mM NAD groups. Additionally, the intensity of OMP, a marker for mature OSNs, was significantly increased in the NAD groups, in particular in the second week (Fig. 2a,b).

**Fig. 3:**
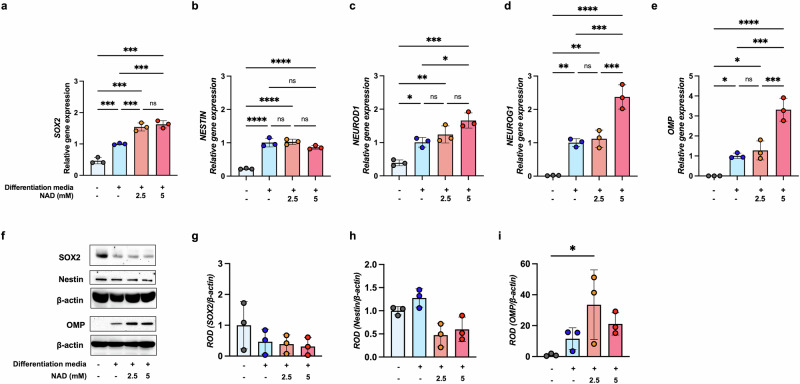
NAD enhances expression of neuronal stem cell and olfactory sensory neuron markers in human olfactory stem cells. Experiments were conducted under four distinct conditions: neuronal differentiation media (**–**), differentiation media (+) without NAD, and differentiation media (+) with 2.5 mM or 5.0 mM NAD for 1 week. **a–e**, Gene expression analysis showed increased levels of *SOX2*, *NESTIN*, *NEUROD1*, *NEUROG1*, and *OMP* following NAD treatment. **f–i**, Western blot analysis revealed elevated expression of olfactory sensory neuron marker (OMP) with NAD treatment at day 14. ROD, relative optical density. *P < 0.05, **P < 0.01, ***P < 0.001, ****P < 0.0001, ns, nonspecific. *n* = 3 in each group.

Moreover, PCR analyses assessing gene expression at 1 week after differentiation related to neuronal stem cells and both immature and mature OSNs, including *SOX2*, *NESTIN*, *NEUROD1*, *NEUROG1*, and *OMP* (Fig. 3a–e), revealed that the differentiated cell group exhibited significantly higher expression of all five genes compared with the undifferentiated hOSC group. As described earlier, *SOX2* and *NESTIN* are established markers of neuronal stem cells. *NEUROD1* and *NEUROG1* are markers of neuronal regeneration, whereas *OMP* is a marker of mature OSN^29^. Notably, *NEUROD1, NEUROG1*, and *OMP* demonstrated elevated expression levels in the NAD treatment groups compared with the untreated group in the PCR analysis after 1 week of differentiation. Western blot analysis was conducted to further validate these findings at the protein level at 2 weeks of differentiation (Fig. 3f–i). A statistically significant increase in OMP expression was observed in the NAD-treated groups, indicating enhanced maturation of OSNs. Although no statistically significant differences were observed in nestin and SOX2 protein levels, a slight decrease was observed in the NAD-treated groups at 2 weeks of differentiation, suggesting a transition from neuronal stem cells to OSNs.

### Intranasal administration of NAD improved olfactory function in anosmia mice

**Fig. 4:**
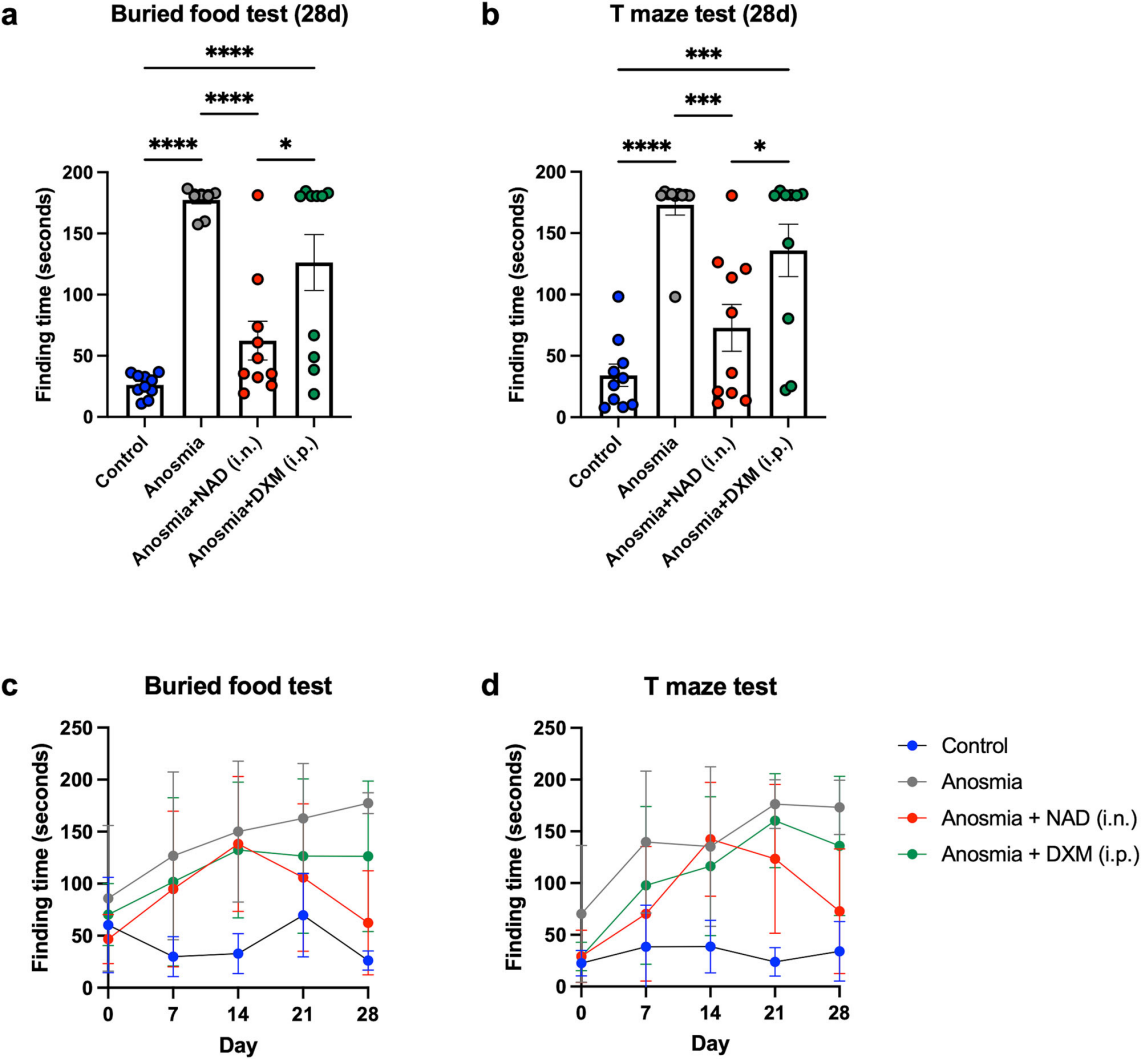
Intranasal administration of NAD improved olfactory function in mice with anosmia. Between-group comparison of the results in the buried food (part **a**) and T-maze tests (part **b**) on day 28. **c**,**d**, The trends of results on days 0, 7, 14, 21, and 28. DXM, dexamethasone; i.n., intranasal; i.p., intraperitoneal. *P < 0.05, **P < 0.01, ***P < 0.001, ****P < 0.0001. *n* = 10 in each group.

In vivo behavioral tests were conducted to evaluate the recovery of olfactory function in mice. Results from the open field and T-maze tests indicated that, compared with the DXM and anosmia groups, the olfactory function in the NAD group significantly improved by day 28 (Fig. 4a,b). We noted that olfactory function steadily declined until day 14 across the anosmia, NAD, and DXM groups. However, improvement was observed in the NAD group by days 21 and 28 (Fig. 4c,d). These behavioral tests demonstrated that i.n. administration of NAD effectively restored olfactory function in the ZnSO_4_-induced anosmia mouse model. Furthermore, i.n. treatment with NAD outperformed systemic DXM, the current conventional therapy for olfactory loss.

To validate that the effects observed in the NAD and DXM groups were attributable to their regenerative potential rather than direct effects on olfactory function, additional behavioral tests were conducted in control mice treated with NAD or DXM. No significant differences in finding time were observed across all groups, indicating that NAD and DXM administration alone did not influence olfactory function (Supplementary Fig. 3). To compare the efficacy of i.n. versus i.p. NAD administration, we evaluated olfactory function on day 28. Intranasal NAD treatment demonstrated significantly greater improvement compared with i.p. administration, as evidenced by shorter finding times in both behavioral tests (Supplementary Fig. 4). These results suggest that i.n. delivery of NAD provides superior therapeutic benefits for olfactory function recovery compared with systemic administration.

### Intranasal administration of NAD restored olfactory epithelium in anosmia mice

**Fig. 5:**
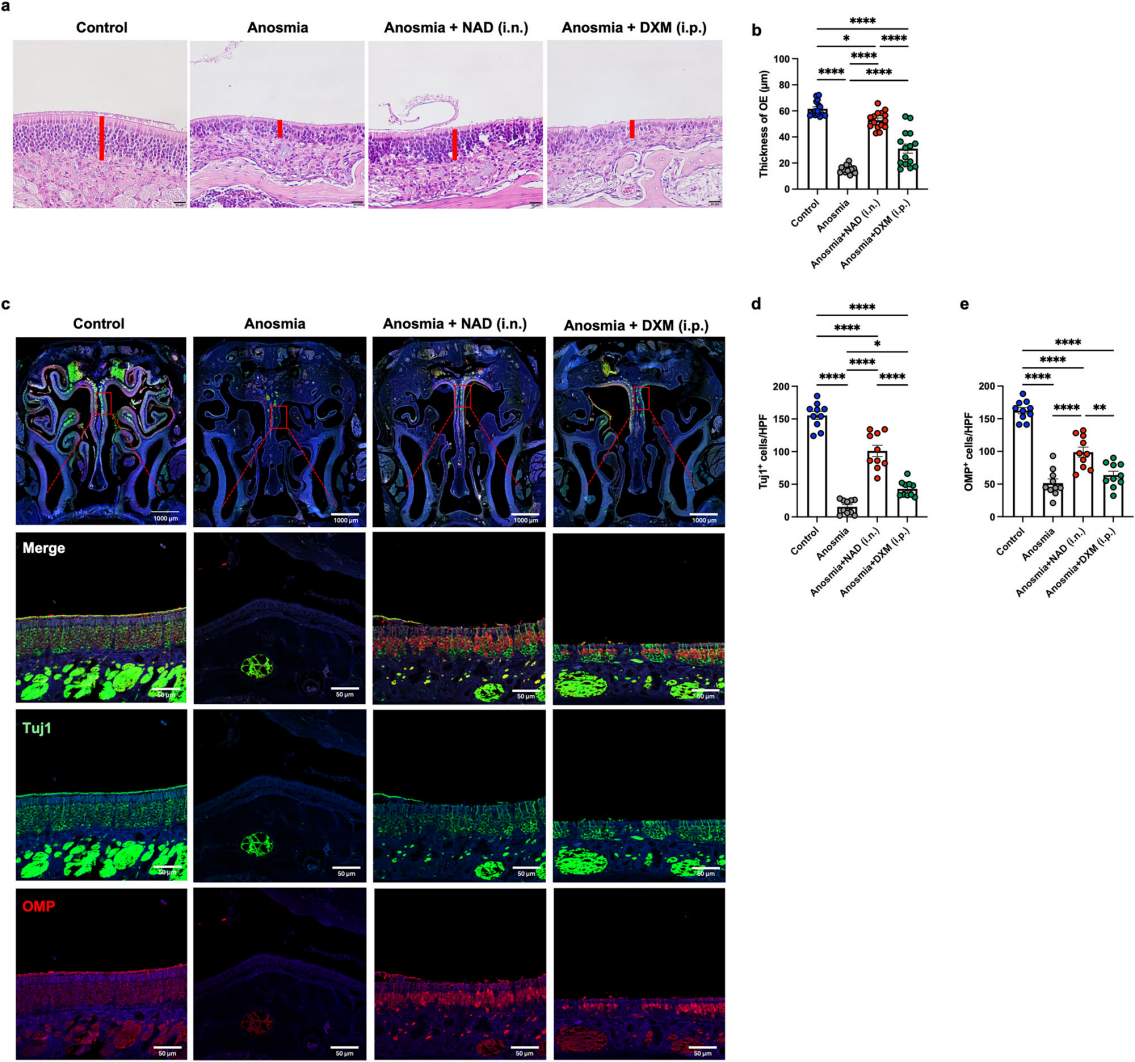
Histological analyses of mouse olfactory epithelium. **a**,**b**, Hematoxylin and eosin staining of the olfactory mucosa in the nasal cavity showed significantly greater olfactory epithelium (OE) thickness (indicated by red lines) in the NAD group compared with the dexamethasone (DXM) and anosmia groups (*P* < 0.0001), although it remained lower than the control group (*P* < 0.05). **c–e**, Immunofluorescence staining revealed that the mean Tuj1^+^ cell count per high power field (HPF, at 400× magnification) in the NAD group was significantly higher than in the anosmia, DXM, and control groups. Furthermore, the number of olfactory marker protein (OMP)-positive cells in the NAD group was significantly higher than that in the anosmia (*P* < 0.0001) and DXM (*P* < 0.01) groups. These findings indicate that intranasal NAD treatment promotes both immature and mature olfactory sensory neuron regeneration more effectively than systemic DXM treatment. i.n., intranasal; i.p., intraperitoneal. *P < 0.05, **P < 0.01, ***P < 0.001, ****P < 0.0001, *n* = 10 in each group. Scale bar, 20 µm (part **a**) and 50 µm (part **c**).

The thickness of the OE layer was measured on H&E-stained slides (Fig. 5a,b). Mice in the control group exhibited the thickest mean OE layer, measuring 61.6 ± 5.8 μm. By contrast, the anosmia group had the thinnest mean OE layer at 15.2 ± 2.8 μm. The OE thickness in the DXM group varied widely, from 15.2 μm to 55.7 μm, with a mean of 31.0 ± 13.5 μm, significantly greater than the anosmia group (*P* < 0.0001) but considerably lower than the control group (*P* < 0.0001). The NAD group had a mean OE thickness of 52.9 ± 6.5 μm, significantly greater than the DXM and anosmia groups (*P* < 0.0001) but less than that the control group (*P* < 0.05).

The quantification of immature and mature OSNs was performed using confocal microscopy (Fig. 5c). The number of Tuj1^+^ cells, representing immature OSNs, exhibited a pattern consistent with the observed effects on OE thickness. The control group demonstrated the highest mean Tuj1^+^ cell count per HPF at 145.6 ± 12.3, whereas the anosmia group presented the lowest mean count at 29.4 ± 10.8 cells/HPF. The DXM group showed a mean Tuj1^+^ cell count of 46.8 ± 15.2 cells/HPF, which was higher than the anosmia group. The NAD group demonstrated a mean Tuj1^+^ cell count of 92.4 ± 18.5 cells/HPF, which was significantly higher than both the anosmia group and the DXM group (*P* < 0.0001) (Fig. 5d). These results suggest that NAD treatment exerts a more pronounced effect on promoting neuronal regeneration compared with DXM. Although Tuj1 is an established marker for immature OSNs, it is also expressed in certain mature OSNs during the differentiation process of hOSCs into OSNs^30^. The number of OMP^+^ cells, mature OSNs, also followed a trend similar to the number of OE thickness and Tuj1^+^ cells. The control group had the highest mean number of OMP^+^ cells/HPF at 162.4 ± 15.0, whereas the anosmia group had the lowest mean number at 51.4 ± 20.4 cells/HPF. The DXM group had a mean OMP^+^ number of 63.8 ± 18.7 cells/HPF, which did not differ significantly from the anosmia group; by contrast, the NAD group had a mean OMP^+^ cell count of 98.8 ± 24.6 cells/HPF, which was significantly higher than the anosmia group (*P* < 0.0001) and DXM group (*P* < 0.01) (Fig. 5e).

### Comprehensive analysis of gene expression via RNA-seq in mice and validation in human OSCs

**Fig. 6:**
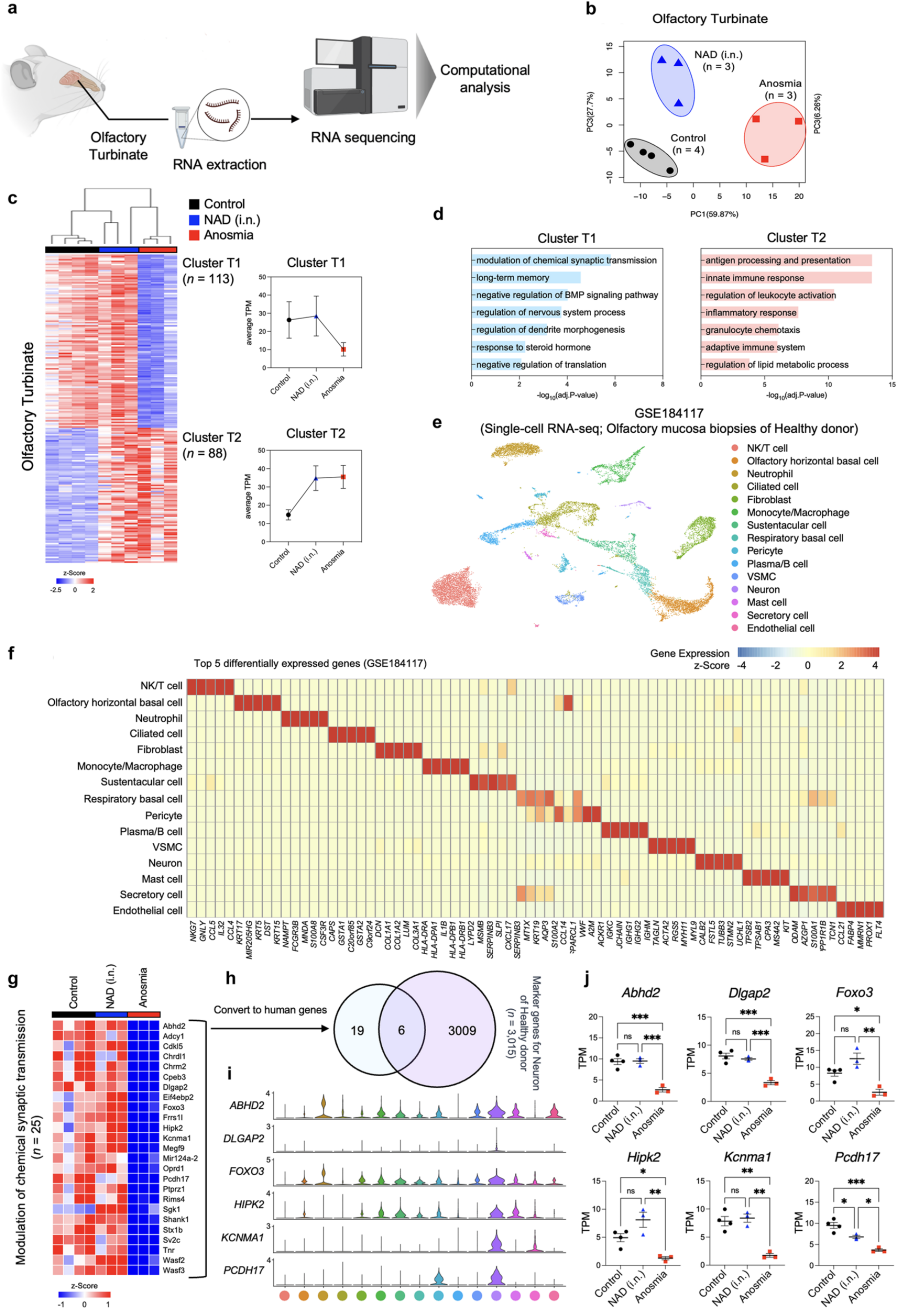
Comprehensive analysis of gene expression via RNA-sequencing analyses. **a**, Schematic representation of sample preparation from olfactory turbinate, followed by RNA extraction and computational analysis via RNA-sequencing. **b**, Principal component (PC) analysis plots displaying sample clustering from olfactory turbinate in control, anosmia, and NAD-treated mice. **c**, Gene expression in mouse olfactory turbinate. Left: heatmap illustrating the expression levels of differentially expressed genes (DEGs) meeting specific criteria (false discovery rate < 0.05; twofold difference in expression level; average transcript per million (TPM) > 2). *K*-means clustering (*k* = 2) analysis of 201 DEGs across three groups, with values presented as *z*-score. Right: line graph depicting characteristic gene expression patterns for each cluster. **d**, Gene Ontology (GO) analysis was performed for Cluster T1 and T2 genes. **e**, Uniform manifold approximation and projection visualization of single-cell RNA-seq data (GSE184117) for 15,997 single cells obtained from olfactory mucosa biopsies of healthy donors, categorizing cells into clusters based on gene expression profiles. **f**, Heatmap showing top 5 DEGs across various cell types from olfactory mucosa biopsies of healthy donors, with gene expression intensity indicated by color gradient from low (green) to high (red). Values are *z*-scores. **g**, Heatmap illustrating the expression patterns of genes related to modulation of chemical synaptic transmission within Cluster T1. **h**, Venn diagram illustrating the overlap between genes related to modulation of chemical synaptic transmission and molecular markers of human olfactory neurons. **i**, Violin plots depicting the distribution of normalized expression levels for selected genes related to the modulation of chemical synaptic transmission across different cell types, color-coded to match the cell types identified in part **d**. **j**, Graphs presenting TPM values for candidate genes. BMP, bone morphogenetic protein; i.n., intranasal; NK, natural killer; ns, nonspecific; VSMC, vascular smooth muscle cell. *P < 0.05, **P < 0.01, ***P < 0.001.

Bulk RNA-seq was performed on olfactory turbinate tissues from the control, NAD, and anosmia groups to delineate their distinct and shared gene expression profiles (Fig. 6a). Principal component analysis, utilizing the expression data of all genes, distinctly classified the groups according to their respective conditions (Fig. 6b). Two unique gene expression patterns were identified, resulting in 201 DEGs. Of these, 113 DEGs (cluster T1) were upregulated in the control and NAD groups, whereas 88 (cluster T2) were downregulated exclusively in the control group (Fig. 6c). Gene Ontology (GO) analysis was used to decipher the molecular mechanisms and biological functions associated with genes in each identified cluster. The GO terms “modulation of chemical synaptic transmission”, “regulation of nervous system process”, and “response to steroid hormone” were associated with cluster T1 (Fig. 6d, left panel). By contrast, the GO terms “antigen processing and presentation”, “innate immune response”, and “inflammatory response” GO terms were associated with cluster T2 (Fig. 6d, right panel).

Cellular deconvolution was subsequently applied using a validated public scRNA-seq dataset to identify candidate genes expressed in olfactory receptor neurons within the pool of identified genes^26^. A total of 15,997 single cells from olfactory mucosa biopsies of healthy donors revealed diversity across 15 cell types, including neuronal populations, each distinguished by a unique profile of the top 5 DEGs (Fig. 6e,f). Twenty-five genes implicated in the modulation of synaptic chemical transmission were upregulated after NAD stimulation (Fig. 6g). Intersecting these genes with neuronal marker genes acquired using scRNA-seq analysis identified candidate genes (Fig. 6h). Six candidate genes — *ABHD2*, *DLGAP2*, *FOXO3*, *HIPK2*, *KCNMA1*, and *PCDH17* — were found to be expressed in OSNs (Fig. 6i). The expression of these genes was significantly upregulated in the NAD group compared with the anosmia group, with their expression levels being restored to levels similar to those observed in the control group (Fig. 6j).

**Fig. 7:**
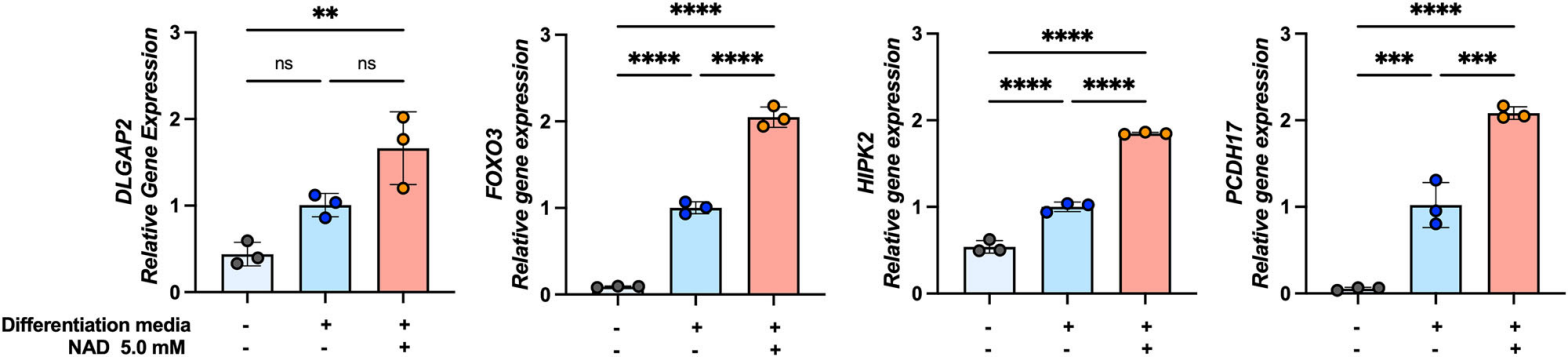
In human olfactory stem cells, PCR analyses of *DLGAP2*, *FOXO3*, *HIPK2*, and *PCDH17* upregulated in the RNA-sequencing analysis. The expression levels of *DLGAP2*, *FOXO3*, *HIPK2*, and *PCDH17* were significantly increased by NAD treatment. **P < 0.01, ***P < 0.001, ****P < 0.0001. ns, nonspecific. *n* = 3 in each group.

These six genes identified from RNA-seq data were further validated using PCR analyses in hOSCs. PCR analysis of the *KCNMA1* gene showed no significant differences between the NAD-treated group and the untreated group (data not shown), whereas the *ABHD2* gene was excluded from the analysis owing to its predominant expression in oligodendrocytes in brain tissue and its undetermined function^31^. The expression levels were significantly higher in the differentiated cell group compared with the undifferentiated hOSC group in all four remaining genes: *DLGAP2*, *FOXO3*, *HIPK2*, and *PCDH17* (Fig. 7). Moreover, the NAD treatment group exhibited significantly higher levels of *DLGAP2*, *FOXO3, HIPK2*, and *PCDH17* gene expression compared with the untreated group. Among these genes, *DLGAP2* and *PCDH17* are closely associated with neuronal regeneration, synapse formation, and axonal growth^32–35^. NAD treatment in hOSCs significantly increased the protein expression of DLGAP2 and PCDH17, as demonstrated by immunofluorescence staining (Supplementary Fig. 5), whereas the expression of FOXO3 was not well detected in hOSCs by immunofluorescence staining (data not shown).

**Fig. 8:**
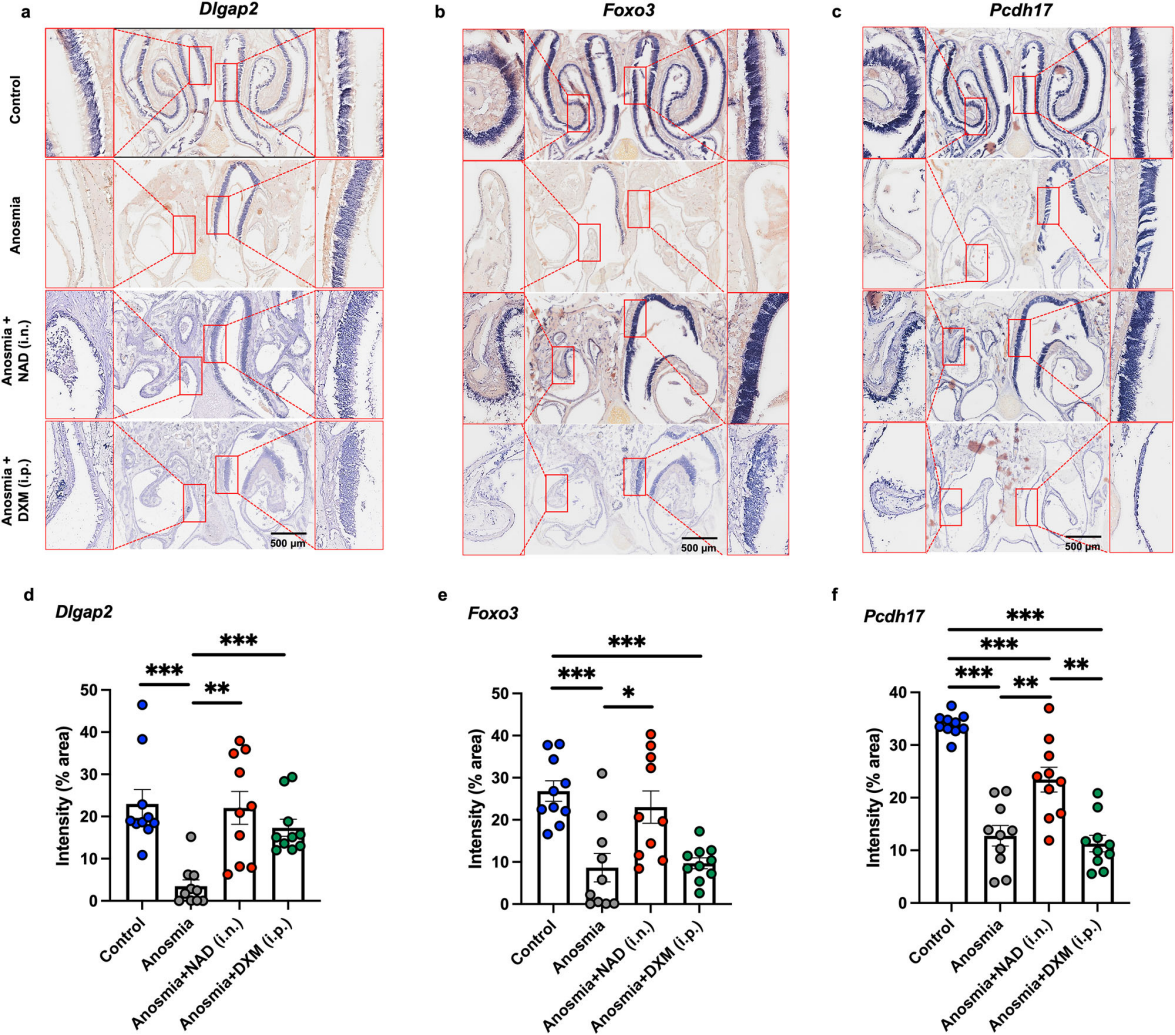
In vivo validation of transcriptomic targets using RNA in situ hybridization in an anosmia mouse model. Representative RNA in situ hybridization images and quantitative analysis of signal intensity for *Dlgap2* (parts **a** and **d**), *Foxo3* (parts **b** and **e**), and *Pcdh17* (parts **c** and **f**) in the murine olfactory epithelium. The anosmia group exhibited marked downregulation of all three target genes compared with the control group. Intranasal NAD treatment significantly restored the mRNA expression levels of *Dlgap2*, *Foxo3*, and *Pcdh17*, demonstrating therapeutic efficacy of NAD. Red dashed boxes indicate the magnified regions of the olfactory epithelium. DXM, dexamethasone; i.n., intranasal; i.p., intraperitoneal. *P < 0.05, **P < 0.01, ***P < 0.001. Scale bar, 500 µm.

To further validate the transcriptomic analysis results identified in our sequencing data within a complex tissue environment, we performed ISH analysis using an in vivo mouse model. We examined the spatial expression patterns of three key DEGs — *Dlgap2*, *Foxo3*, and *Pcdh17* — in olfactory tissue sections following ZnSO_4_-induced injury and subsequent treatment with NAD (i.n.) or DXM (i.p.) (Fig. 8). In the PBS control group, strong mRNA expression signals for *Dlgap2*, *Foxo3*, and *Pcdh17* were predominantly localized to the OE, confirming their abundant expression in healthy tissue. By contrast, the ZnSO_4_-induced anosmia group exhibited marked disruption of the epithelial architecture and a significant reduction in signal intensity for all three genes (*P* < 0.0001), reflecting extensive tissue damage and transcriptional downregulation caused by the injury. Intranasal treatment with NAD significantly restored the expression levels of these molecular markers. The ISH images revealed a reconstituted epithelial layer with robust mRNA signals comparable to those observed in the control group. Quantitative analysis of signal intensity confirmed that NAD treatment resulted in statistically significant upregulation of *Dlgap2*, *Foxo3*, and *Pcdh17*, compared with the anosmia group (*P* < 0.0001). These in vivo findings provide direct tissue-level confirmation of our transcriptomic data, demonstrating that i.n. NAD treatment effectively preserves the molecular profile of the OE and promotes tissue recovery following injury.

## Discussion

In this study, we explored the therapeutic efficacy of NAD in olfactory dysfunction. Our results demonstrated that i.n. administration of NAD significantly improved olfactory function. This improvement was accompanied by enhanced neurogenesis and maturation of OSNs, as evidenced by behavioral tests and histological analyses. These findings are consistent with previous studies that highlighted the neuroprotective and neuroregenerative properties of NAD in various neurodegenerative disease models^15,36^.

Regarding the observation that stem cell markers, as well as immature and mature OSN markers, were all upregulated, we attribute this phenomenon to the dual role of NAD in promoting stem cell function and enhancing neuronal differentiation. NAD appears to activate pathways that maintain the stemness of hOSCs while simultaneously facilitating their differentiation into OSNs. This interpretation aligns with findings from previous studies on the role of NAD in neuronal development^37–39^. Notably, although markers for immature (Tuj1^+^) and mature (OMP^+^) OSNs were both elevated after NAD treatment, their expression levels exhibited temporal differences, suggesting a sequential progression from immature to mature OSNs (Fig. 2). SOX2 is widely recognized as a marker of pluripotent neural stem cells and is expressed in OSCs (horizontal and globose basal cells) and sustentacular cells in the OE^27,40^, and nestin is expressed in undifferentiated neural stem cells during development^28^. In hOSCs treated with NAD, the expression of neuronal stem cell marker (nestin) and OSN markers (Tuj1 and OMP) increased at the end of first and second weeks. The increase in OMP expression from 1 week to 2 weeks, alongside the maintenance of Tuj1 expression, represents a characteristic pattern in the olfactory system. Our results align with the patterns observed in OSN development, as described in ref. ^41^, wherein the increase in OMP expression, indicating mature OSNs, from 1 to 2 weeks, along with sustained Tuj1 expression, reflects the continuous processes of neurogenesis and maturation within the olfactory system. Additionally, studies by Gavid et al. have demonstrated that Tuj1 is expressed in neurons transitioning from an immature to a mature state and persists throughout the maturation process^30,41^. By contrast, the expression of the pluripotent stem cell marker (SOX2) increased at the end of first week but decreased at the end of second week, suggesting that SOX2^+^ cells decreased as they underwent neuronal differentiation in OSCs. These findings suggest that NAD treatment induced neuronal differentiation of SOX2^+^ pluripotent OSCs and promoted their differentiation into nestin^+^ neuronal stem cells followed by immature and mature OSNs in the process of differentiation into neurons. Furthermore, NAD increased the expression of genes associated with neuronal differentiation (*SOX2*, *NESTIN*, *NEUROD1*, *NEUROG1*, and *OMP*) and the protein levels of SOX2, nestin, and OMP. Although in vitro cultured hOSCs provide a reliable platform for investigating OSN differentiation and NAD effects^42,43^, they may not fully recapitulate the complexity of in vivo conditions. Advanced experimental systems, such as organoids or ex vivo models, could offer more comprehensive insights and more accurately simulate the native environment. Future investigations utilizing these sophisticated models would further validate and extend the findings of the present study.

In anosmia mice model, i.n. administration of NAD significantly improved olfactory function and enhanced neurogenesis and maturation of OSNs, as evidenced by behavioral tests and histological analyses (OE thickness and expression of Tuj1 and OMP). These findings are consistent with previous studies that highlighted the neuroprotective and neuroregenerative properties of NAD in various neurodegenerative disease models^14,15^. In both hOSCs and mouse, significant increase in Tuj1 expression by NAD treatment means that NAD may increase immature OSNs during the recovery phase following injury and the increase in OMP expression suggests that NAD promotes OSNs maturation.

Of particular interests were the RNA-sequencing results of olfactory turbinate tissues in mice, which indicated upregulation of critical genes involved in neuronal development, chemical synaptic transmission, and olfactory sensory function in NAD-treated mice. In RNA-seq analysis, 25 genes involved in the modulation of synaptic chemical transmission were identified as upregulated after NAD stimulation. By intersecting these genes with human neuronal marker genes, six potential genes were significantly overexpressed in the NAD group compared with the anosmia group. To further elucidate the mechanism, NAD-treated hOSCs showed elevated expression of *DLGAP2, FOXO3, HIPK2*, and *PCDH17* compared with the untreated group. All these genes are expressed in the OSNs of human OE^26^. Previous studies have associated these genes with cell survival, protection against oxidative stress, and nerve or cell regeneration^35,44–46^. *DLGAP2* encodes essential elements of postsynaptic scaffolding proteins crucial for synaptic morphogenesis and function^33,47–49^. It is implicated in regulating age-related neurodegeneration, cognitive decline, and Alzheimer disease^32^. On the basis of the findings of this study, NAD administration likely mitigated olfactory neuronal degeneration by increasing *DLGAP2* expression. Forkhead box transcription factors (FOXOs) such as FOXO3 have a crucial role in enhancing the ability of adult hematopoietic and neural stem cells to regenerate by imparting resistance to oxidative stress^50–52^. Additionally, NAD has been shown to promote neuronal survival under oxidative stress conditions through mechanisms involving protein kinase B and mitochondrial membrane potential, along with *FOXO3a*^45^. This observation that NAD treatment increased FOXO3 expression in this study suggests that NAD may prevent loss of olfactory function by enhancing the ability of OSNs to withstand oxidative stress. *HIPK2* encodes a serine–threonine kinase that is evolutionarily conserved^53^. HIPK2 is abundantly expressed in both the central and peripheral nervous systems, and its genetic ablation in mice leads to neurological abnormalities. Moreover, *HIPK2* regulates the expression of genes involved in antioxidant production and response to reactive oxygen species^54^. Similar to the FOXO3 gene, it is hypothesized that HIPK2, upregulated by NAD, promoted the survival of OSNs and facilitated the differentiation of OSCs into OSNs in environments with high oxidative stress. Procadherin genes, including *PCDH8*, *10*, *17*, *18*, and *19*, regulate cell motility^55,56^. *PCDH17* mediates collective axon extension by recruiting actin regulator complexes to interaxonal contacts^34^. Intranasal administration of NAD increased *PCDH17* expression, suggesting enhanced axonal extension during OSN regeneration. Further studies using transgenic mice will be needed to elucidate the precise mechanisms by which NAD affects olfactory nerve regeneration, which may provide valuable insights into the pathological processes associated with anosmia. Among these genes, *DLGAP2* and *PCDH17*, which are involved in axonal regeneration, synapse formation and neural connectivity, neuroplasticity, and neural network modulation, are suggested to be the most crucial genes^32–35^ (Supplementary Fig. 5).

To validate our transcriptomic findings at the tissue level, we performed ISH analysis in mouse models of olfactory injury. Although transcriptomic analysis provides comprehensive screening of gene expression changes, tissue-level validation is essential to confirm the spatial localization and physiological relevance of DEGs within the intact tissue architecture. Our ISH results demonstrated that the expression of *Dlgap2*, *Foxo3*, and *Pcdh17* was markedly reduced in the damaged OE of anosmic mice but was significantly restored following i.n. NAD treatment (Fig. 8). These findings provide direct tissue-level evidence that NAD treatment upregulates these neuroregenerative factors at the mRNA level specifically within the regenerating OE, confirming that the transcriptomic changes we observed reflect biologically meaningful alterations in gene expression within the target tissue. The spatial distribution of these markers within the OE further supports their functional roles in olfactory neuroregeneration. Notably, the concordance among our transcriptomic data, in vitro protein expression in cultured hOSCs, and in vivo tissue-level gene expression in the mouse OE validates the molecular mechanisms of NAD-mediated olfactory recovery and supports their translational relevance.

In conclusion, this study provides promising results for the potential use of NAD as a therapeutic agent to treat olfactory dysfunction by comprehensively analyzing its remarkable effects on in vitro neuronal differentiation in hOSCs and olfactory function, histology, and gene expression, using an anosmia mouse model. NAD may promote neurogenesis and maturation of OSNs and restore olfactory function by activating genes involved in synaptic transmission and neuronal survival, such as *DLGAP2* and *PCDH17*. These results will be an important basis for exploring clinical applications of NAD.

## Supplementary information

Supplementary Information
