# Supplementary material for: Therapeutic effect of intranasal nicotinamide adenine dinucleotide in the restoration of olfactory dysfunction
Source: Exp Mol Med. 2026 Jul 1;58(7):2155–67. doi: 10.1038/s12276-026-01761-9 (PMC13434466; doi:10.1038/s12276-026-01761-9)
Supplement: Supplementary file 1 [file 12276_2026_1761_MOESM1_ESM.pdf]

Online Supplementary Table and Figures

Supplementary Table 1. Primers used in PCR analysis to determine the extent of differentiation of hOSCs treated with NAD

Genes	Forward (5′-3′)	Reverse (5′-3′)
DLGAP2	CCTCTGACATCACCTCCACCAT	TCACCGAGATCAGAGGCTTGGA
FOXO3	TCTACGAGTGGATGGTGCGTTG	CTCTTGCCAGTTCCCTCATTCTG
HIPK2	AGCGTCATCACCATCAGCAGTG	AGTCGTGGACTGTGACACAGCT
PCDH17	CAAAGGCTCCTGCTGTGACATG	GACCAAGCACTCGGCATTCATC
SOX2	GCTACAGCATGATGCAGGACCA	TCTGCGAGCTGGTCATGGAGTT
NESTIN	TCAAGATGTCCCTCAGCCTGGA	AAGCTGAGGGAAGTCTTGGAGC
NEUROD1	GGTGCCTTGCTATTCTAAGACGC	GCAAAGCGTCTGAACGAAGGAG
NEUROG1	GCCTCCGAAGACTTCACCTACC	GGAAAGTAACAGTGTCTACAAAGG
OMP	CAAGGAGGACTCGGATGCCATA	TCGCCAAAGGTGACGAGGAAGT
GAPDH	AGGTCGGTGTGAACGGATTT	TGTAGACCATGTAGTTGAGG

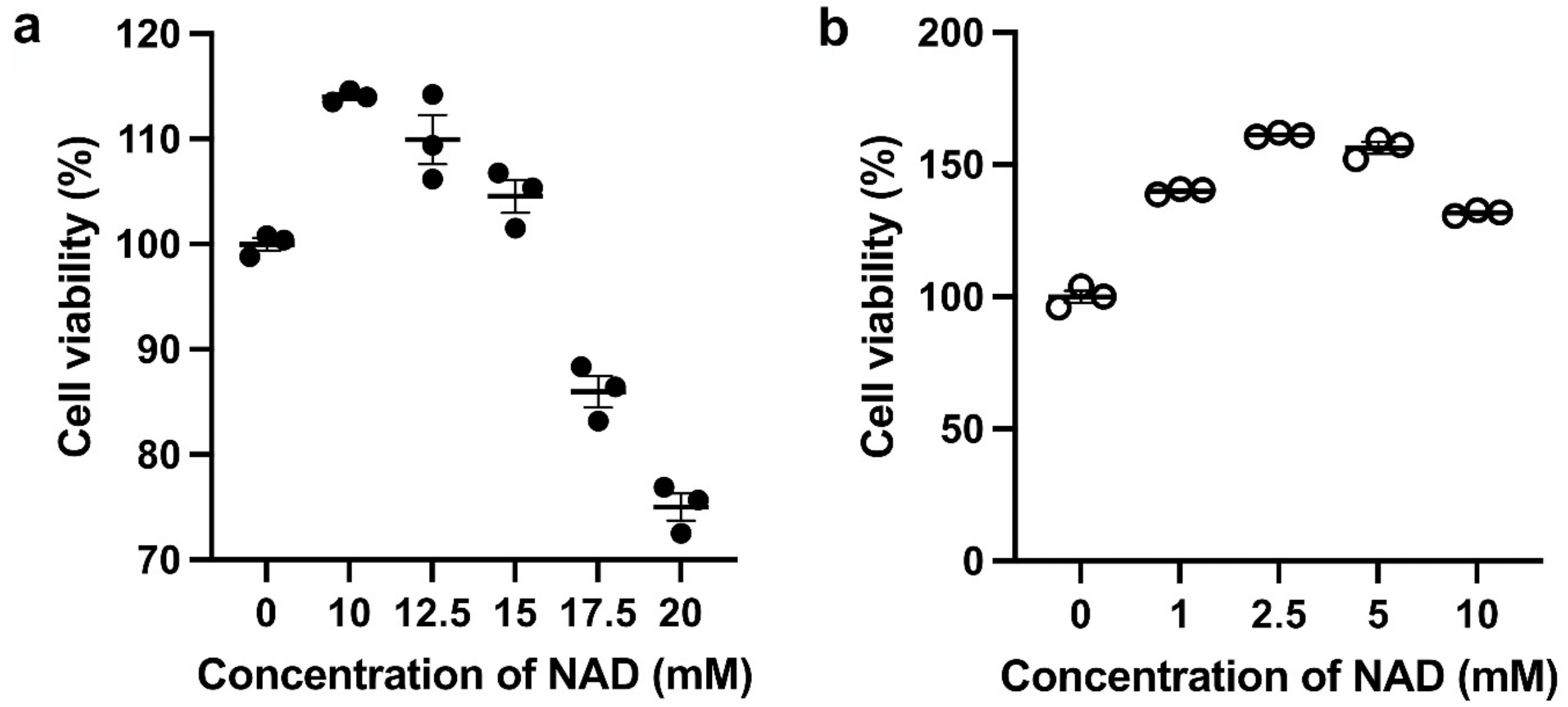

Supplementary Figure 1. Cell viability assay results using CCK-8, confirming the optimal NAD dose. Two sets of NAD concentrations were tested: **a** 0, 10, 12.5, 15, 17.5, and 20 mM; and **b** 0, 1, 2.5, 5, and 10 mM (n = 3 in each group).

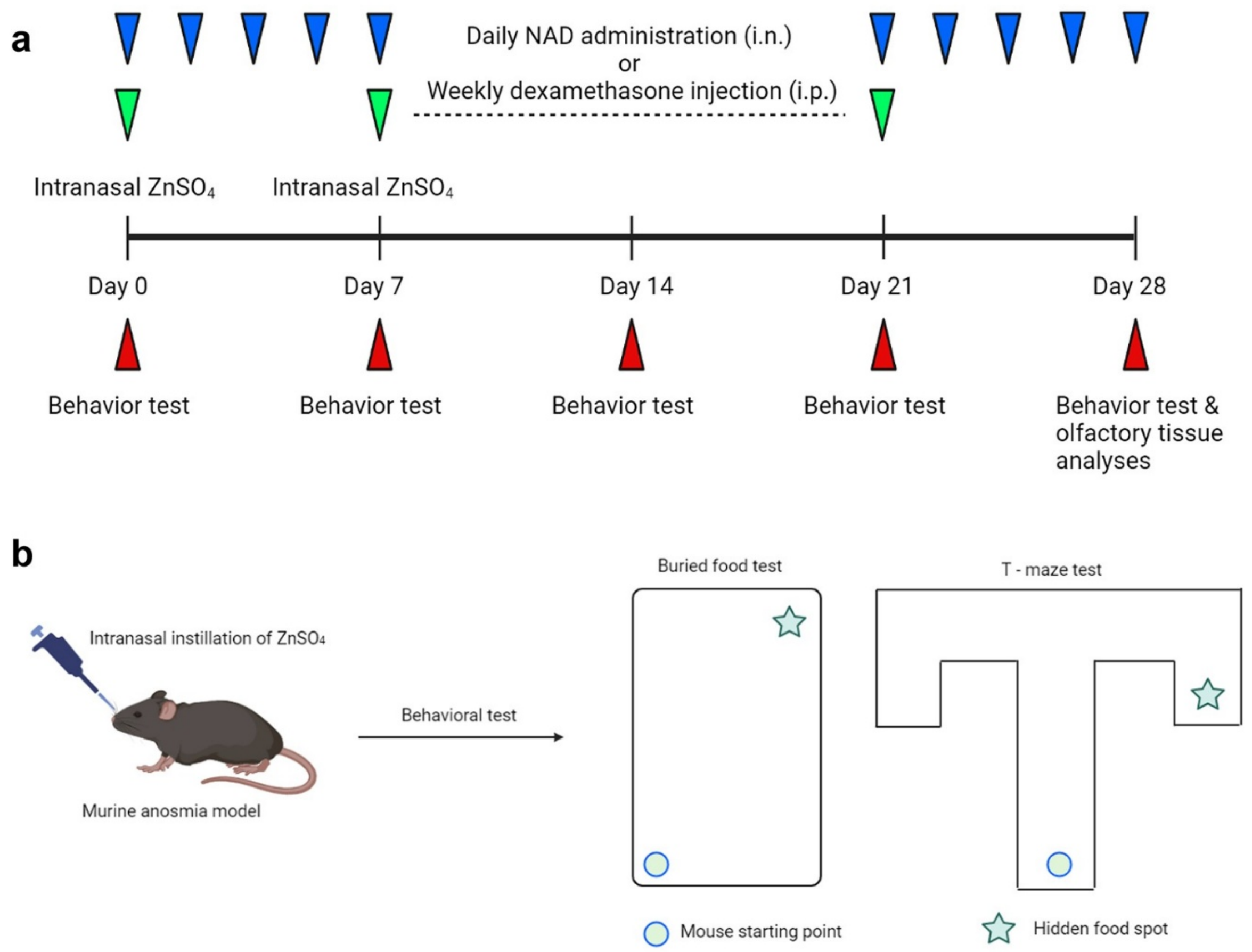

Supplementary Figure 2. Schematic illustration of in vivo experiment and behavioral test. **a** The anosmia mice model was generated by subjecting mice to weekly intranasal (i.n.) administration of $ZnSO_4$. The treatment group received daily i.n. administration of nicotinamide adenine dinucleotide (NAD), whereas the positive control group received weekly intraperitoneal (i.p.) injection of dexamethasone (DXM). Behavioral tests were performed once every week for one month, and mice were euthanized for tissue analyses on day 28. **b** Behavioral tests (buried food and T-maze tests) of the mouse models.

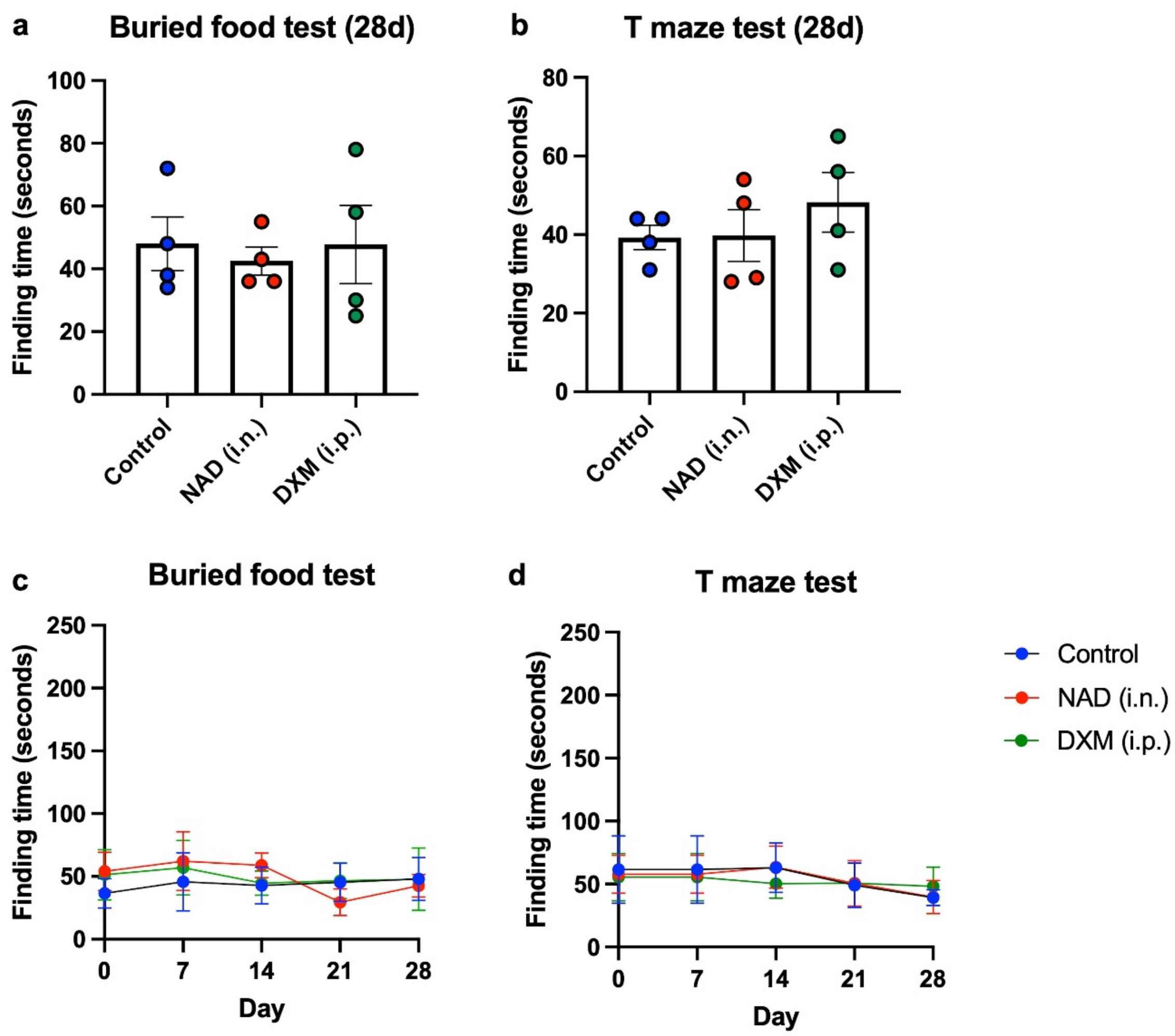

Supplementary Figure 3. Behavioral assessment of olfactory function to evaluate the effects of intranasal administration of nicotinamide adenine dinucleotide (NAD) and intraperitoneal injection of dexamethasone (DXM) in control mice. a, b The buried food test and T-maze test performed on day 28 showed no significant differences in finding time among groups, indicating that NAD (intranasal, i.n.) and DXM (intraperitoneal, i.p.) administration in control mice did not affect olfactory function. **c, d** Time-course analysis of the buried food test and T-maze test showed consistent results over the 28-day period.

Data are presented as mean ± SEM. n = 4 per group.

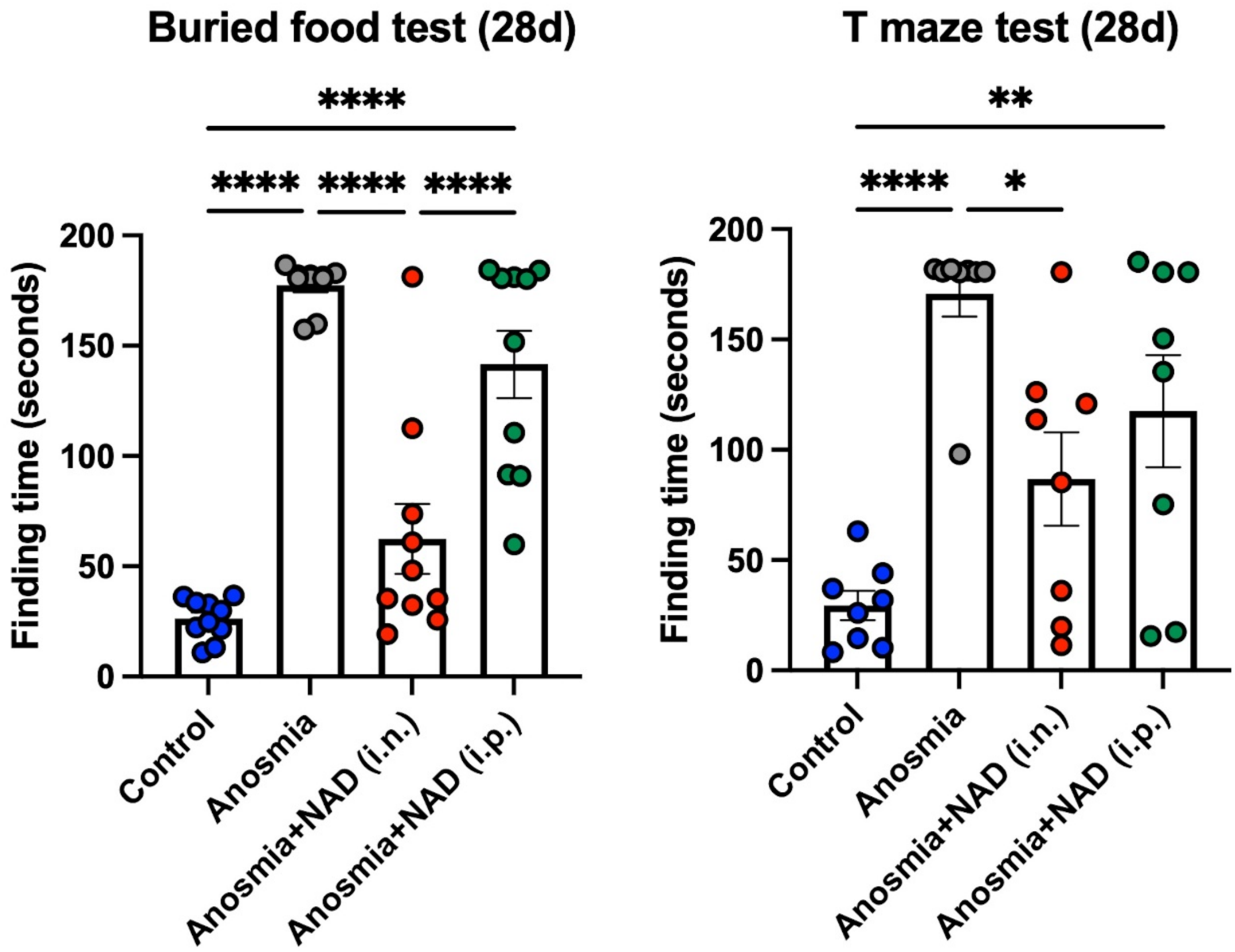

Supplementary Figure 4. Intranasal nicotinamide adenine dinucleotide (NAD) administration is more effective than systemic administration in restoring olfactory function. Compared to intranasal (i.n.) NAD treatment, systemic injection (intraperitoneal, i.p.) of NAD did not significantly improve olfactory function in mice with anosmia.

* *P* < 0.05, ** *P* < 0.01, *** *P* < 0.001, **** *P* < 0.0001, ns, non-specific. Data are presented as mean ± SEM. n = 8 in each group.

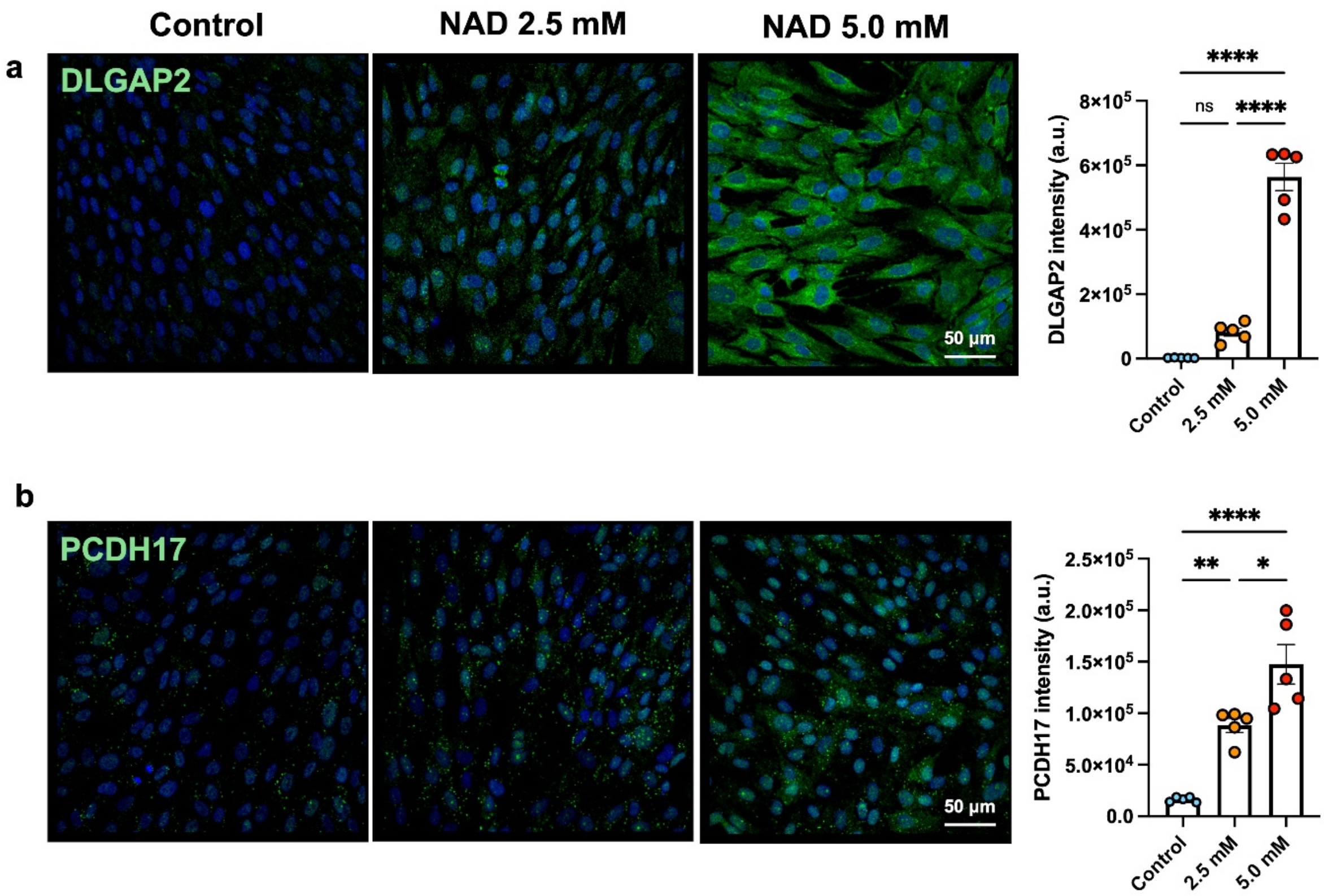

Supplementary Figure 5. Immunostaining validation of neuronal differentiation-related genes in human olfactory stem cells (hOSCs). a, b Immunofluorescence staining showed that the intensities of DLGAP2 and PCDH17 significantly increased in hOSCs treated with nicotinamide adenine dinucleotide (NAD, 2.5 mM and 5.0 mM) compared to the control group, suggesting their involvement in neuronal differentiation and regeneration.

a.u.: arbitrary units

** $P < 0.05$, ** $P < 0.01$, *** $P < 0.001$, **** $P < 0.0001$, n = 4 in each group. Scale bar, 50 μm.
